# Effect of NK cell receptor genetic variation on allogeneic stem cell transplantation outcome and in vitro NK cell cytotoxicity

**DOI:** 10.1038/s41598-024-78619-5

**Published:** 2024-11-06

**Authors:** Julia Nihtilä, Leena Penna, Urpu Salmenniemi, Maija Itälä-Remes, Rachel E. Crossland, David Gallardo, Katarzyna Bogunia-Kubik, Piotr Lacina, Maria Bieniaszewska, Sebastian Giebel, Katariina Karjalainen, Farhana Jahan, Erja Kerkelä, Kati Hyvärinen, Satu Koskela, Jarmo Ritari, Jukka Partanen

**Affiliations:** 1 Research and Development, Finnish Red Cross Blood Service, Helsinki, Finland; 2 Advanced Cell Therapy Centre, Finnish Red Cross Blood Service, Vantaa, Finland; 3 Comprehensive Cancer Center, Stem Cell transplantation Unit, Helsinki University Hospital, Helsinki, Finland; 4 University of Helsinki, Helsinki, Finland; 5 Turku University Hospital, Turku, Finland; 6 Translational and Clinical Research Institute, Faculty of Medical Science, Newcastle University, Newcastle upon Tyne, UK; 7 Department of Hematology, ICO Girona, Girona, Spain; 8 Laboratory of Clinical Immunogenetics and Pharmacogenetics, Hirszfeld Institute of Immunology and Experimental Therapy, Polish Academy of Sciences, Wroclaw, Poland; 9 Department of Hematology and Transplantology, Medical University of Gdansk, Gdansk, Poland; 10 Department of Bone Marrow Transplantation and Hematology-Oncology, Gliwice Branch, Maria Skłodowska-Curie Memorial Cancer Centre and Institute of Oncology, Gliwice, Poland; 11 Blood Service Biobank, Finnish Red Cross Blood Service, Vantaa, Finland

**Keywords:** Leukaemia, Immunogenetics

## Abstract

Natural killer (NK) cells recognize and may kill malignant cells via their cell surface receptors. Killer cell immunoglobulin-like receptor (KIR) genotypes of donors have been reported to adjust the risk of relapse after allogeneic stem cell transplantation (HSCT), particularly in patients with acute myeloid leukemia. To test whether non-KIR NK cell receptors have a similar effect, we screened 1,638 genetic polymorphisms in 21 non-KIR NK cell receptor genes for their associations with relapse and graft-versus-host disease (GVHD) after HSCT in 1,491 HSCT donors (from Finland, the UK, Spain, and Poland), divided into a discovery and replication cohort. Eleven polymorphisms regulating or located in CD226, CD244, FCGR3A, KLRD1, NCR3, and PVRIG were associated with the risks for relapse and GVHD. These associations could not be confirmed in the replication cohort. Blood donor NK cells carrying alleles showing genetic protection for relapse had a higher in vitro NK cell killing activity than non-carriers whereas those with alleles genetically protective for GVHD had lower cytotoxicity, potentially indicating functional effects. Taken together, these results show no robust effects of genetic variation in the tested non-KIR NK cell receptors on the outcome of HSCT.

**Supplementary Information:**

The online version contains supplementary material available at 10.1038/s41598-024-78619-5.

## Introduction

Natural killer (NK) cells are cytotoxic immune cells that can kill malignant cells^1,2^. NK cell function is regulated by a complex network of cell receptor–ligand interactions, both inhibitory and activating, which may mask the effect of single factors. While killer cell immunoglobulin-like receptors (KIRs) appear to be amongst the key factors defining NK cell activity in allogeneic hematopoietic stem cell transplantation (HSCT), other NK cell receptors also regulate the activity^1,2^. These receptors include, for example, natural killer group 2, natural cytotoxicity receptors, immunoglobulin-like transcripts, CD226, and 2B4 ^3^. NK cells can detect and kill malignant cells, for example, with missing or disturbed human leukocyte antigen (HLA) class I expression^4^.

NK cells are the first lymphocyte subset appearing after HSCT, their count reaching the normal levels in about three months post-transplant^5,6^. The beneficial effect of NK cell activation in HSCT was introduced by the studies of Ruggeri and co-workers^7^. They showed that acute myeloid leukemia (AML) patients with a specific HLA–KIR ligand mismatch with the donor were protected from relapse after HSCT. They furthermore showed that in the HLA–KIR ligand mismatched setting, NK cells were activated and killed the malignant cells^8^. The association between the KIR ligand mismatch and reduced risk for relapse has been found in many independent studies^9–11^, however, not in a recent large study^12^. Cooley and co-workers^13,14^, as well as many others^15–17^, have shown that selecting the donors with KIR genetic haplotype B, that is, chromosomes containing a higher number of activating KIR gene types, improved relapse-free survival after HSCT. These studies have led to suggestions that it may be advantageous to select the HSCT donors according to their KIR genotypes for patients with AML. Whether the higher killing activity of the KIR haplotype B carriers can be shown in in vitro experiments is still unclear.

The relative importance of different NK cell receptors to the functional properties of NK cells or to the HSCT cell donor selection is currently not established. The receptors most likely act in a complex crosstalk. For example, NKp46 was reported to avert graft-versus-host disease (GVHD) by killing immature dendritic cells^18^ and CD226 has been shown to be involved in the development of T-cell mediated GVHD in mice^19^. Hence, genetic variation in the non-KIR NK cell receptor genes could also be associated with the clinical outcomes of HSCT, particularly with relapse and GVHD. Indeed, for example, variation in the NKG2D gene has been reported to be associated with HSCT outcome^20^ .

The ability to efficiently kill malignant cells makes NK cells an interesting candidate for novel cell therapies in settings other than HSCT^21^. In the past few decades, NK cell therapy has grown into an active field in cancer immunotherapy research. The sensitivity of the target cells to NK cell killing and therapeutic effects appears complex. The effects of cell donor genetics, such as the KIR genotype, are obvious^14^. On the other hand, Sheffer and co-workers^22^ found that NK cell-sensitive targets typically had a mesenchymal cell type signature, with high B7-H6, low HLA-E, and low antigen presenting machinery expression levels. Hence, optimal selection criteria for cell therapy donors not only depend on the donor profile but also on the expected target cell types.

To better understand the role of various non-KIR NK cell receptors in HSCT and cell therapy, we investigated the association of 1,638 genetic polymorphisms in NK cell receptor genes with relapse and GVHD after HSCT in 1,491 donors from Finland, the UK, Spain, and Poland. We focused on the donor NK cell receptor genotypes only, as we can expect that it primarily is the donor rather than the patient, whose NK cell killing activity is most important after HSCT. To find complementary functional evidence, we determined the effects of the associated genetic polymorphisms on the in vitro killing activity of NK cells isolated from genotyped blood donors. Although interesting association trends were found, we conclude that no statistically significant associations could be confirmed, and that there are no strong effects mediated by the non-KIR NK cell receptor gene polymorphisms.

## Results

### Association of NK cell receptor gene polymorphisms with HSCT outcomes

**Fig. 1:**
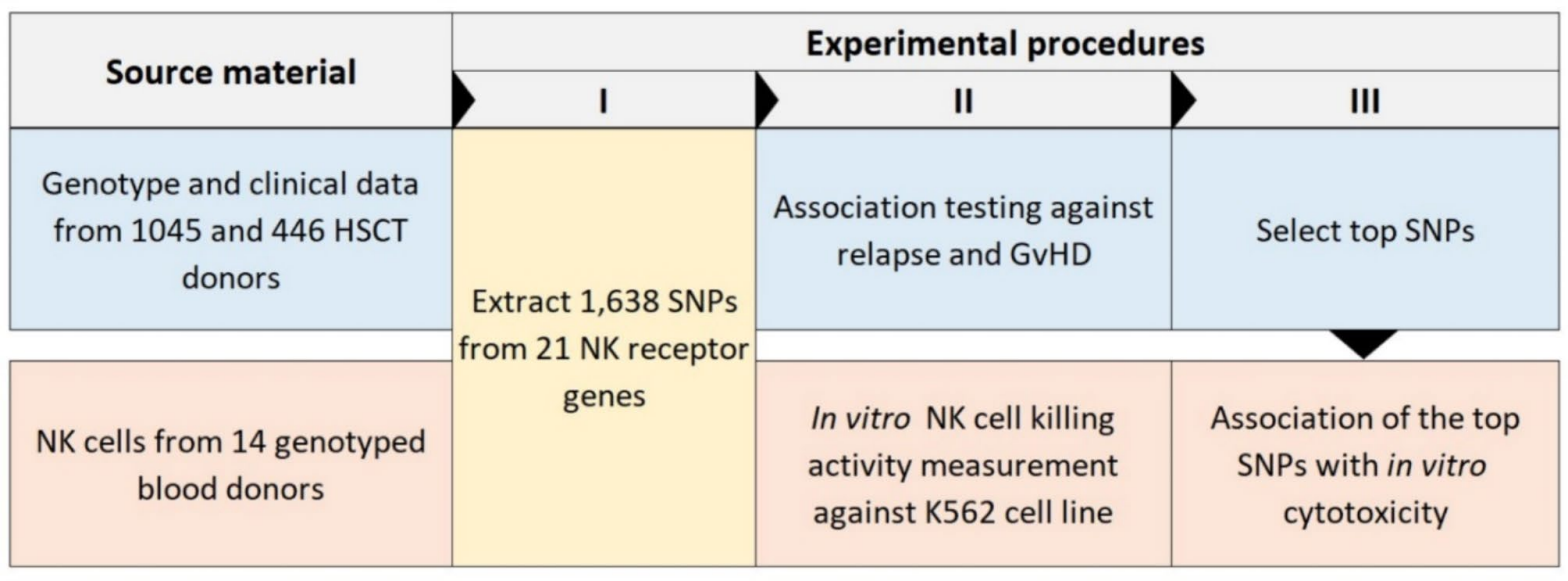
Overview of the workflow.

**Fig. 2:**
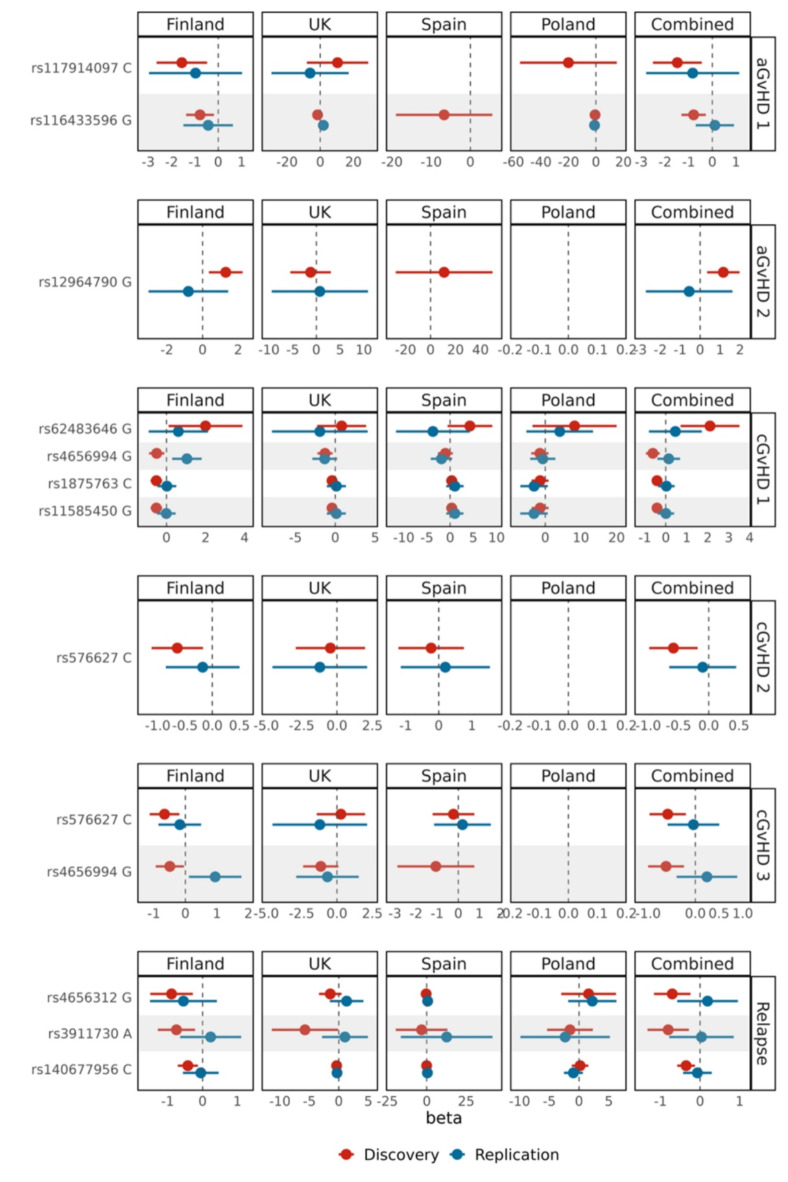
Beta values of NK cell receptor gene polymorphisms associated with acute GVHD, chronic GVHD, and relapse in study populations. aGvHD 1 indicates acute GVHD grade I-IV vs. grade 0; aGvHD 2 indicates acute GVHD grade III-IV vs. grade 0; cGvHD 1 indicates chronic GVHD limited, extensive, grade unknown vs. grade 0; cGvHD 2 indicates chronic GVHD extensive vs. grade 0, cGvHD 3 indicates chronic GVHD extensive, grade unknown vs. grade 0; relapse indicates relapse yes vs. no. Results for the subpopulation Poland are missing, when the phenotype was unavailable. Results for the subpopulation Spain are missing when all individuals had the same genotype. The error bars signify the confidence intervals (CI).

Demographic data of the study cohorts are shown in Table 1. It is of note that there were HSCTs from four different populations and from multiple HSCT units. An overview of the workflow is shown in Fig. 1. We analyzed the genetic association of 1,638 NK cell receptor gene polymorphisms with acute GVHD, chronic GVHD, and relapse in 1,491 HSCT donors divided into a discovery (*n* = 1,045 donors) and replication (*n* = 446 donors) cohort. Eleven polymorphisms provided evidence for statistical associations (Fig. 2). All results of the association analyses can be found in the Supplementary Material (Tables S2–S15) online. Minor allele frequencies and genotype counts for selected polymorphisms can be found in the Supplementary Material online (Supplementary Tables S35 and S36).

**Table 1:** Demographics of the HSCT study populations.

	Finland		UK		Spain		Poland	
	Discovery	Replication	Discovery	Replication	Discovery	Replication	Discovery	Replication
Number of HSCT donors, n	572	193	237	115	179	93	57	45
HSCT time, years	1993–2011	2011–2018	1984–2011	2011–2017	2000–2009	2009–2014	2019–2021	2021–2022
Missing, n (%)	17 (3)	8 (4)	0 (0)	0 (0)	0 (0)	0 (0)	0 (0)	0 (0)
Recipient age in years, median (range)	50 (18–70)	52 (20–69)	41 (19–65)	53 (20–72)	50 (18–69)	52 (20–72)	50 (20–73)	52 (20–73)
Missing, n (%)^a^	0 (0)	0 (0)	0 (0)	0 (0)	2 (1)	1 (1)	0 (0)	1 (2)
Donor age in years, median (range)	44 (16–73)	39 (16–68)	37 (12–63)	34 (19–71)	46 (4–78)	51 (17–75)		
Missing, n (%)^a^	278 (49)	0 (0)	6 (3)	7 (6)	47 (26)	11 (12)	57 (100)	45 (100)
Donor-recipient gender, n (%)								
Male–male	175 (31)	76 (39)	94 (40)	52 (45)	60 (34)	31 (33)	22 (39)	17 (38)
Male–female	143 (25)	49 (25)	60 (25)	34 (30)	42 (23)	20 (22)	10 (18)	10 (22)
Female–male	117 (20)	29 (15)	47 (20)	12 (10)	42 (23)	25 (27)	14 (25)	8 (18)
Female–female	120 (21)	39 (20)	35 (15)	16 (14)	35 (20)	17 (18)	11 (19)	10 (22)
Missing	17 (3)	0 (0)	1 (0)	1 (1)	0 (0)	0 (0)	0 (0)	0 (0)
Stem cell source, n (%)								
Peripheral blood	320 (56)	156 (81)	120 (51)	108 (94)	169 (94)	89 (96)	56 (98)	45 (100)
Bone marrow	248 (43)	37 (19)	116 (49)	1 (1)	10 (6)	4 (4)	0 (0)	0 (0)
Both	2 (0)	0 (0)	0 (0)	0 (0)	0 (0)	0 (0)	1 (2)	0 (0)
Missing	2 (0)	0 (0)	1 (0)	6 (5)	0 (0)	0 (0)	0 (0)	0 (0)
Donor type, n (%)								
Sibling	470 (82)	76 (39)	145 (61)	27 (23)	179 (100)	93 (100)	37 (65)	28 (62)
Register	102 (18)	112 (58)	92 (39)	88 (77)	0 (0)	0 (0)	1 (2)	1 (2)
Haplo	0 (0)	5 (3)	0 (0)	0 (0)	0 (0)	0 (0)	19 (33)	16 (36)
Missing	0 (0)	0 (0)	0 (0)	0 (0)	0 (0)	0 (0)	0 (0)	0 (0)
Conditioning regimen, n (%)								
Myeloablative	434 (76)	129 (67)	107 (45)	15 (13)	78 (44)	35 (38)	33 (58)	36 (80)
Reduced intensity	136 (24)	53 (27)	130 (55)	100 (87)	95 (53)	58 (62)	23 (40)	8 (18)
Other	2 (0)	11 (6)	0 (0)	0 (0)	0 (0)	0 (0)	0 (0)	1 (2)
Missing	0 (0)	0 (0)	0 (0)	0 (0)	6 (3)	0 (0)	1 (2)	0 (0)
GvHD prophylaxis, n (%)^b^								
1	429 (75)	142 (74)	55 (23)	7 (6)	102 (57)	49 (53)	36 (63)	25 (56)
2	26 (5)	18 (9)	112 (47)	81 (70)	20 (11)	3 (3)	1 (2)	0 (0)
3	72 (13)	27 (14)	0 (0)	10 (9)	15 (8)	17 (18)	1 (2)	1 (2)
4	1 (0)	4 (2)	0 (0)	2 (2)	8 (4)	1 (1)	19 (33)	19 (42)
5	2 (0)	0 (0)	47 (20)	2 (2)	0 (0)	0 (0)	0 (0)	0 (0)
Missing	42 (7)	2 (1)	23 (10)	13 (11)	34 (19)	23 (25)	0 (0)	0 (0)
Donor-recipient CMV combination, n (%)								
Donor positive, Recipient negative	28 (5)	8 (4)	34 (14)	8 (7)	18 (10)	5 (5)	7 (12)	1 (2)
Other combinations	178 (31)	133 (69)	200 (84)	99 (86)	117 (65)	64 (69)	50 (88)	44 (98)
Missing	366 (64)	52 (27)	3 (1)	8 (7)	44 (25)	24 (26)	0 (0)	0 (0)
Donor with at least one HLA-C1 allele, n (%)	498 (87)	165 (85)	204 (86)	101 (88)	135 (75)	77 (83)	44 (77)	40 (89)
Missing	0 (0)	0 (0)	0 (0)	0 (0)	0 (0)	0 (0)	0 (0)	0 (0)
Donor with at least one HLA-C2 allele, n (%)	445 (78)	157 (81)	210 (89)	101 (88)	157 (88)	74 (80)	50 (88)	37 (82)
Missing	0 (0)	0 (0)	0 (0)	0 (0)	0 (0)	0 (0)	0 (0)	0 (0)
HLA-match, n (%)								
10/10	513 (90)	174 (90)	147 (62)	85 (74)	151 (84)	78 (84)	35 (61)	27 (60)
Other	15 (3)	19 (10)	41 (17)	21 (18)	6 (3)	7 (8)	17 (30)	16 (36)
Missing	44 (8)	0 (0)	49 (21)	9 (8)	22 (12)	8 (9)	5 (9)	2 (4)
aGvHD, n (%)								
Grade 0	374 (65)	92 (48)	85 (36)	32 (28)	122 (68)	55 (59)	32 (56)	29 (64)
Grade I-II	130 (23)	72 (37)	121 (51)	58 (50)	33 (18)	23 (25)	19 (33)	16 (36)
Grade III-IV	64 (11)	28 (15)	28 (12)	1 (1)	24 (13)	15 (16)	6 (11)	0 (0)
Grade unknown	0 (0)	1 (1)	0 (0)	0 (0)	0 (0)	0 (0)	0 (0)	0 (0)
Missing	4 (1)	0 (0)	3 (1)	24 (21)	0 (0)	0 (0)	0 (0)	0 (0)
cGvHD, n (%)								
Grade 0	253 (44)	89 (46)	81 (34)	20 (17)	81 (45)	34 (37)	23 (40)	22 (49)
Yes, classification unknown	1 (0)	0 (0)	20 (8)	0 (0)	0 (0)	0 (0)	12 (21)	8 (18)
Limited	105 (18)	28 (15)	56 (24)	32 (28)	16 (9)	8 (9)	0 (0)	0 (0)
Extensive	171 (30)	68 (35)	46 (19)	29 (25)	41 (23)	15 (16)	0 (0)	0 (0)
Missing	42 (7)	8 (4)	34 (14)	34 (30)	41 (23)	36 (39)	22 (39)	15 (33)
Relapse, n (%)								
Yes	201 (35)	66 (34)	80 (34)	23 (20)	52 (29)	16 (17)	9 (16)	4 (9)
No	364 (64)	127 (66)	156 (66)	74 (64)	127 (71)	75 (81)	48 (84)	41 (91)
Missing	7 (1)	0 (0)	1 (0)	18 (16)	0 (0)	2 (2)	0 (0)	0 (0)
Diagnosis, n (%)^c^								
Acute myeloid leukemia	178 (31)	66 (34)	99 (42)	35 (30)	62 (35)	29 (31)	21 (37)	17 (38)
Acute lymphoblastic leukemia	89 (16)	25 (13)	36 (15)	8 (7)	14 (8)	5 (5)	12 (21)	5 (11)
Myelodysplastic syndrome	51 (9)	21 (11)	11 (5)	12 (10)	21 (12)	9 (10)	3 (5)	4 (9)
Chronic myeloid leukemia	56 (10)	4 (2)	33 (14)	4 (3)	12 (7)	3 (3)	3 (5)	0 (0)
Multiple myeloma	80 (14)	27 (14)	0 (0)	0 (0)	0 (0)	0 (0)	0 (0)	5 (11)
Other	125 (22)	46 (24)	81 (34)	54 (47)	70 (39)	47 (51)	20 (35)	13 (29)

*GvHD* graft-versus-host disease, *aGvHD* acute GvHD, *cGvHD* chronic GvHD, *CMV* cytomegalovirus.

^a^Missing ages were imputed, see Materials and methods.

^b^1: CSA + MTX ± ATG ± MMF or CSA + MTX + steroid or CSA + MTX + ECP or evero + MTX + MMF, 2: CSA ± ATG or CSA + ECP or CSA + steroidi or Tacro ± ATG, 3: CSA + MMF ± ATG or CSA + MMF ± steroid or Tacro + MMF or Tacro + Sirolimus, 4: All combinations with PtCy, 5: Other combinations.

^c^Five most frequent diagnoses.

In the discovery cohort, eQTL allele rs140677956*C of NCR3, eQTL rs3911730*A of CD226, and rs4656312*G of FCGR3A were associated with relapse, with the betas of -0.35 (95% CI: -0.58 to -0.13, covariate-adjusted p-value = 0.002), -0.80 (95% CI: -1.33 to -0.28, covariate-adjusted p-value = 0.003), and − 0.70 (95% CI: -1.16 to -0.24, covariate-adjusted p-value = 0.003), respectively. The alleles reduced the risk for relapse in the entire discovery cohort and in each subpopulation except for rs140677956 and rs4656312 in the Polish population. None of these associations, however, could be confirmed in the replication cohort, with the beta of -0.07 (95% CI: -0.43 to 0.29, covariate-adjusted p-value = 0.71) for rs140677956*C, 0.04 (95% CI: -0.78 to 0.85, covariate-adjusted p-value = 0.93) for rs3911730*A, and 0.19 (95% CI: -0.58 to 0.95, covariate-adjusted p-value = 0.63) for rs4656312*G. None of these associations passed multiple testing correction (Supplementary Table S15 online).

**Fig. 3:**
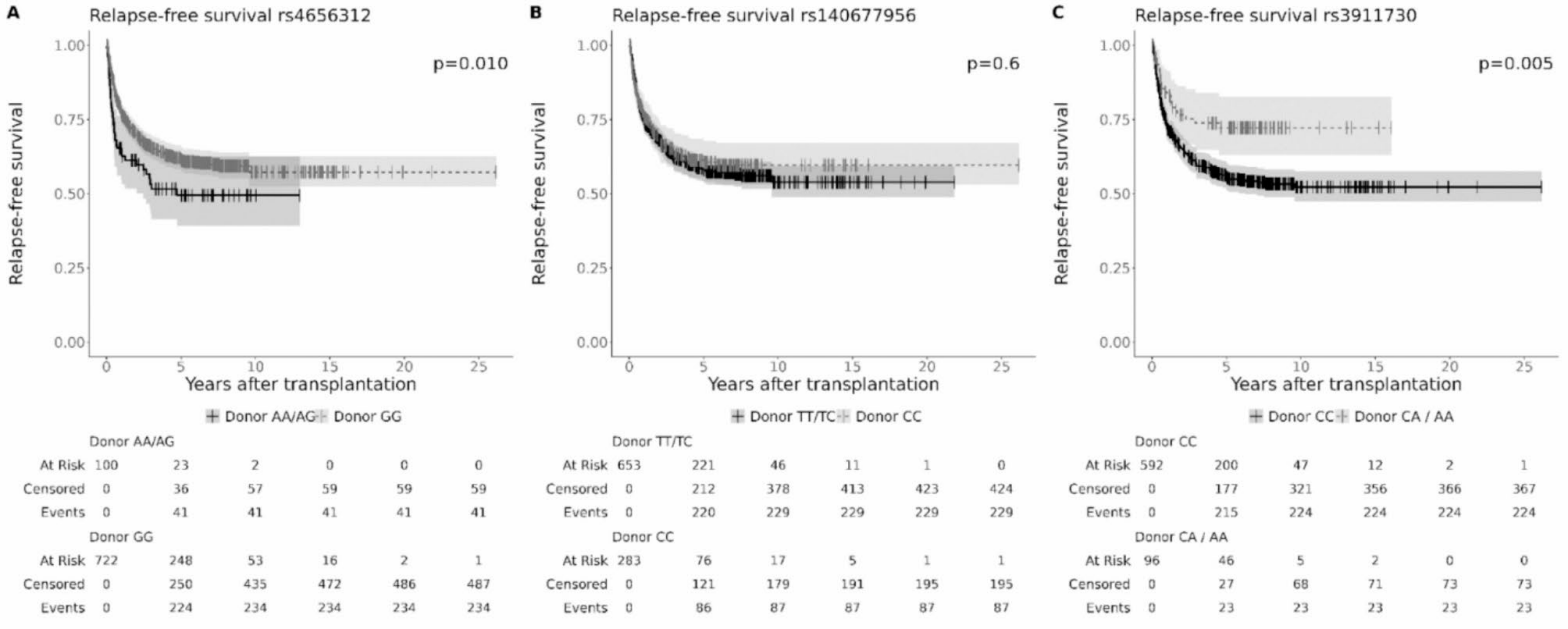
Kaplan-Meier analysis of relapse-free survival in the discovery cohort. (**A**) HSCT donor rs4656312*GG genotype compared to the *AA + *GA genotype. (**B**) HSCT donor rs140677956*CC genotype compared to the *TT + *TC genotype. (**C**) HSCT donor rs3911730*AA + *CA genotypes compared to the * CC genotype.

Figure 3 shows the Kaplan-Meier univariate survival analysis of relapse-free survival (RFS) in relation to the three relapse-protective polymorphisms in the discovery cohort. Cox regression multivariate analyses in the discovery cohort (Supplementary Tables S29 – S31 online) showed a hazard ratio of 0.79 (95% CI: 0.58–1.06 p-value = 0.11, that is, 21% reduction in the rate of RFS), for the rs140677956*CC genotype, compared to the *TT + *TC genotype, a hazard ratio of 0.54 (95% CI: 0.33–0.88. p-value = 0.013, a 46% reduction in the rate of RFS), for the rs3911730*AA + *CA genotypes, compared to the *CC genotype, and a hazard ratio of 0.51 (95% CI: 0.34–0.74 p-value < 0.001, a 49% reduction in the rate of RFS), for the rs4656312*GG genotype, compared to the *AA + *GA genotype. However, the results could not be confirmed in the replication cohort (Supplementary Figures S2 – S4 and Supplementary Tables S32 – S34 online).

The eQTL allele rs116433596*G of CD244, and rs117914097*C, of KLRD1 were associated with a lower risk of acute GVHD (grade I-IV vs. grade 0) with the ORs of 0.45 (95% CI 0.27–0.76, covariate-adjusted p-value = 0.003) and 0.22 (95% CI 0.08–0.64, covariate-adjusted p-value = 0.005), respectively, in the discovery cohort. It is of note that in the discovery subpopulation from the UK, the OR was to the opposite direction. These associations could not be confirmed in the replication cohort (OR 1.11, 95% CI 0.49–2.50, covariate-adjusted p-value = 0.79 for rs116433596; OR 0.44, 95% CI 0.06–3.11, covariate-adjusted p-value = 0.41 for rs117914097) and failed to pass multiple testing correction in the discovery cohort (Supplementary Table S15 online).

The eQTL allele rs12964790*G of CD226 was associated with an increased risk for acute GvHD (grade III-IV vs. grade 0) with the betas of -0.79 (95% CI -1.30 to -0.27, covariate-adjusted p-value = 0.003) and − 1.48 (95% CI -2.51 to -0.45, covariate-adjusted p-value = 0.005) in the discovery cohort, but could not be confirmed in the replication cohort (beta 0.11, 95% CI -0.70 to 0.92, covariate-adjusted p-value = 0.79 for rs116433596; beta − 0.83, 95% CI -2.79 to 1.14, covariate-adjusted p-value = 0.41 for rs117914097) and did not pass multiple testing correction (Supplementary Table S15 online).

For the chronic GVHD (limited, extensive, grade unknown vs. grade 0), three polymorphisms were protective, and one predisposing in the discovery cohort. The eQTL alleles rs4656994*G of CD244, rs11585450*G and rs1875763*C of FCGR3A, and rs62483646*G of PVRIG were associated with betas − 0.61 (95% CI -0.94 to -0.28, covariate-adjusted p-value = 0.0003), -0.42 (95% CI -0.66 to -0.17, covariate-adjusted p-value = 0.0009), -0.41 (95% CI -0.66 to -0.17, covariate-adjusted p-value = 0.0009), and 2.11 (95% CI 0.71 to 3.51, covariate-adjusted p-value = 0.003), respectively. In the Spanish discovery cohort, the betas were to the opposite direction for rs11585450 and rs1875763. These associations did not pass multiple testing correction (Supplementary Table S15 online) and could not be confirmed in the replication cohort (beta 0.14, 95% CI -0.40 to 0.68, covariate-adjusted p-value = 0.61 for rs4656994, beta 0.007, 95% CI -0.37 to 0.40, covariate-adjusted p-value = 0.97 for rs11585450, beta 0.03, 95% CI -0.37 to 0.42, covariate-adjusted p-value = 0.89 for rs1875763, and beta 0.46, 95% CI -0.80 to 1.72, covariate-adjusted p-value = 0.48 for rs62483646).

For the chronic GVHD endpoint extensive vs. no chronic GVHD, one eQTL polymorphism of CD244, rs576627*C, was associated with a reduced risk in the discovery cohort with a beta − 0.58 (95% CI -0.98 to -0.18, covariate-adjusted p-value = 0.004), and for the chronic GVHD endpoint extensive, grade unknown vs. no chronic GVHD, two eQTL polymorphisms of CD244, rs4656994*G and rs576627*C, were associated with a reduced risk in the discovery cohort with betas − 0.62 (95% CI -1.00 to -0.24, covariate-adjusted p-value = 0.001) and − 0.58 (95% CI -1.00 to -0.20, covariate-adjusted p-value = 0.003), respectively. These associations did not pass multiple testing correction (Supplementary Table S15 online) and could not be confirmed in the replication cohort (beta − 0.01, 95% CI -0.65 to 0.45, covariate-adjusted p-value = 0.73 for rs576627 for chronic GVHD extensive vs. no chronic GVHD, beta 0.24, 95% CI -0.40 to 0.88, covariate-adjusted p-value = 0.45 for rs4656994, and beta − 0.04, 95% CI -0.58 to 0.50, covariate-adjusted p-value = 0.89 for rs576627 for chronic GVHD extensive, grade unknown vs. no chronic GVHD).

As the NK cell effect may manifest the strongest in AML, we included the disease group, AML/MDS (*N* = 639) versus the others (*N* = 852), as a covariate in the association analyses. For the polymorphisms, the p-value for this AML covariate ranged between 0.065 and 0.87 (Supplementary Table S14 online), indicating no specific effects in this disease group.

### Polymorphism selection with lasso regression

Lasso regression can perform variable selection by shrinking coefficient estimates to zero, excluding such variables from its models. We provided lasso with the covariates from the association analysis and NK cell receptor gene polymorphism dosages (for all 1,638 polymorphisms) for each of the six endpoints, constructing a model for each endpoint in the discovery and replication cohort. Lasso selected relevant variables from the clinical covariates and polymorphisms to be included in the models (Supplementary Tables S16–S27 online).

Of the polymorphisms with the association with relapse in the discovery cohort, none were included in the lasso models in the discovery cohort (Supplementary Tables S21 and S27 online). Similarly, the lasso models for acute GVHD also contained no polymorphisms with an association to acute GVHD in the HSCT discovery cohort (Supplementary Tables S16 – S17 and S22 – S23 online). Differing from the other endpoints, all polymorphisms with an association to any of the chronic GVHD endpoints in the HSCT discovery cohort were included in the lasso models in the discovery cohort (Supplementary Tables S18 – S20 online), although not in the replication cohort (Supplementary Tables S24 – S26 online), failing to confirm the results of the discovery cohort.

Lasso did provide us with additional evidence for the polymorphism selection since all the chronic GVHD polymorphisms were included in the lasso models. Based on all the analyses described above, we concluded to have sufficient support to test all the eleven polymorphisms with a nominal association with the outcome of HSCT for their effect on NK cell cytotoxicity in vitro.

### Effect of NK cell receptor gene polymorphisms on in vitro NK cell cytotoxicity

**Fig. 4:**
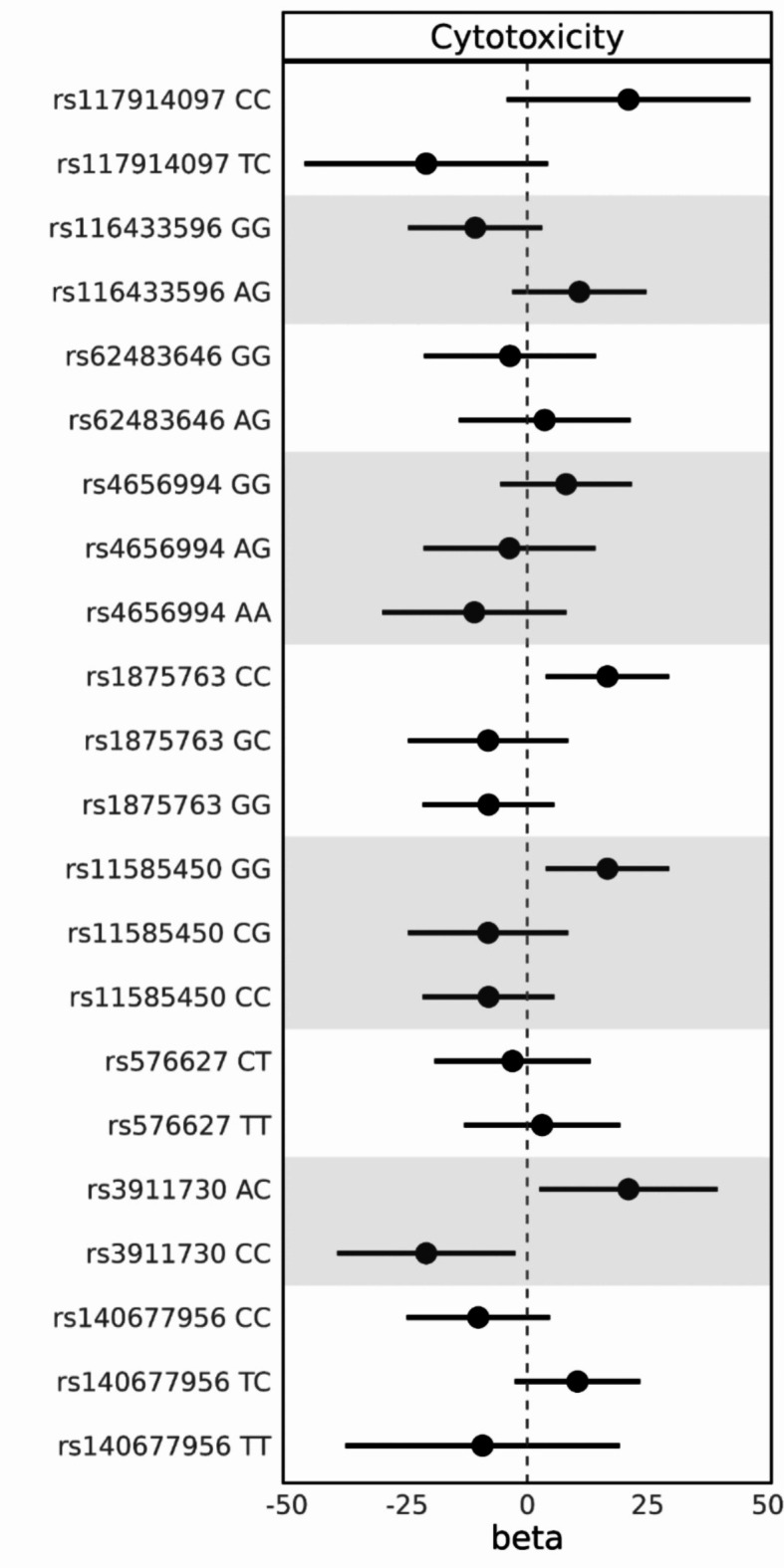
The effect of NK cell receptor gene polymorphisms genotypes on NK cell cytotoxicity in vitro. The NK cell cytotoxicity of the blood donors was analysed using linear mixed-effect models with a random intercept using the lme4 R package. The models included E: T ratios, CMV status, HLA-C genotypes, KIR B content group, and the NK cell receptor gene polymorphisms, as well as a group effect term to connect technical replicates and samples for the random intercept. The error bars signify the confidence intervals (CI).

Using NK cells from 14 genotyped blood donors from the Blood Service Biobank, we analyzed the effect of the eleven NK cell receptor gene polymorphisms, rs116433596, rs4656312, rs4656994, rs11585450, rs1875763, rs576627, rs140677956, rs62483646, rs117914097, rs12964790, rs3911730, on NK cell in vitro killing activity. The results are depicted in Fig. 4 and Supplementary Table S28. Minor allele frequencies and genotype counts for the polymorphisms can be found in the Supplementary Material online (Supplementary Tables S37 and S38).

For the CD226 eQTL polymorphisms, NK cells from blood donors with genotype rs3911730*AC had a higher cytotoxicity than *CC homozygotes (beta 21.0, 95% CI: 2.45–39.6, covariate-adjusted p-value = 0.11), no rs3911730*AA genotypes were present. All blood donors had the same genotype for rs12964790, also in CD226, excluding it from the analysis. NK cells from blood donors with CD244 eQTL polymorphisms rs116433596 *GG and rs576627*CT had a lower cytotoxicity (beta − 10.8, 95% CI: -24.8–3.13, covariate-adjusted p-value = 0.25; beta − 3.08, 95% CI: -19.3–13.1, covariate-adjusted p-value = 0.77; respectively), no homozygotes rs116433596*AA and rs576627*CC were present. NK cells with rs4656994*GG genotype, also an eQTL polymorphism for CD244, had a higher cytotoxicity than the *AA and *AG genotypes (beta 8.06, 95% CI: -5.66–21.8, covariate-adjusted p-value = 0.38).

NK cells from blood donors with rs11585450*GG and rs1875763*CC, both eQTL polymorphisms for FCGR3A, had increased cytotoxicity (beta 16.6, 95% CI: 3.81–29.5, covariate-adjusted p-value = 0.07; beta 16.6, 95% CI: 3.81–29.5, covariate-adjusted p-value = 0.07; respectively) in a recessive manner. All blood donors had the same genotype for rs4656312, a polymorphism in FCGR3A, excluding it from the analysis. NK cells from blood donors with KLRD1 eQTL polymorphism rs117914097*CC had a higher cytotoxicity (beta 21, 95% CI: -4.3–46.3, covariate-adjusted p-value = 0.22) as compared to the *CT heterozygotes, unfortunately, there were no rs117914097*TT homozygotes. Rs140677956*CC, an eQTL polymorphism for NCR3, and rs62483646*GG, an eQTL polymorphism for PVRIG, were associated with a lower cytotoxicity (beta − 10.2, 95% CI: -25.1–4.74, covariate-adjusted p-value = 0.31; beta − 3.6, 95% CI: -21.5–14.3, covariate-adjusted p-value = 0.76; respectively), there were no rs62483646*AA homozygotes among the blood donors.

## Discussion

As there is evidence that HSCTs with donors carrying KIR haplotype B result in a lower risk of AML relapse after HSCT^13,14^, we analyzed the effect of genetic variation in other, non-KIR, NK cell surface receptors on the outcome of HSCT and additionally on the in vitro killing activity of NK cells. We have previously reported that in the Finnish population, partially overlapping with the present Finnish cohort, donor KIR haplotype B defined by KIR2DS2 and KIR2DL2 was associated with a lower risk for relapse^17^. Unfortunately, the genotyping arrays applied to the present samples did not contain sufficiently genetic markers for KIR gene imputation^23^ and no sufficient DNA was available for a targeted, separate KIR typing. Therefore, we were not able to confirm the role of KIR genotype in the entire study cohort or to estimate its effect size as compared to those of other NK cell receptors. We focused on the effects of donor genotypes, as it can be assumed that the NK cell effects on the outcome of HSCT are predominantly mediated by donor NK cells. The number of HSCT donors was relatively high, 1,491, resulting in a sufficient power to detect the strongest, but not minor effects. The HSCTs were from four different populations and retrospective, both facts that may lead to heterogeneity and obvious limitations in available clinical data.

We found weak genetic associations between polymorphisms in the CD226, CD244, FCGR3A, KLRD1, NCR3, and PVRIG genes, and the risks for relapse, acute GVHD, and chronic GVHD in the HSCT discovery cohort but not in the replication cohort. The discrepancies may result from genuine subpopulation or cohort specific differences in HSCT protocols, or they may reflect false positive findings. As the year of transplantation was one of the covariates, the division into the discovery and replication cohorts according to the transplantation year should not explain the results. It is of note that genome-wide association^24–28^ or matching analyses have not pointed to the role of NK cell receptor gene polymorphisms. It was, nevertheless, of interest to find out that the putative protective alleles of GVHD and relapse reported in the present study had effects on NK cell function in vitro. The CD226 allele rs3911730*A protective of relapse in the HSCT discovery cohort was associated with high NK cell in vitro cytotoxicity in the blood donors, and the CD244 alleles rs116433596*G and rs576627*C protective of GVHD were associated with low in vitro cytotoxicity – effects apparently beneficial to the relapse and GVHD risks. These three polymorphisms are eQTLs regulating the expression levels of CD226 and CD244. CD226, encoding the DNAM-1 molecule^29^, mediates immune cell adhesion, regulates NK cell cytotoxicity, and is involved in T-cell mediated acute GVHD in mouse models^19^. CD244 mediates activation, costimulation, and inhibition of NK cell functions^30^. The results can be interpreted to give further support of the role of donor NK cells. However, our NK cell functional approach suffered from a sparse number of study subjects, particularly the number of samples with the minor alleles was low. The NK cells were obtained from apparently healthy blood donors that is both an advantage as they do not have disease or treatment-related heterogeneity, and a disadvantage as their state most likely does not reflect the HSCT setting. We also acknowledge the complex overall regulation of NK cell activity in which demonstrating single molecule effects is difficult.

Nevertheless, it is too early, based on the present results only, to speculate how or even whether the expression level differences could affect the HSCT outcome. In conclusion, our results based on a retrospective, multipopulation HSCT cohort do not indicate strong and robust role in HSCT outcome for the non-KIR NK cell receptors studied in the present study. As our data analyses included, for example, the year of transplantation and graft type as covariates, we have no reason to assume that the differences between discovery and replication cohorts could directly explain our inability to replicate the results of the discovery cohort. More likely the weak borderline evidence points to false positive findings. However, the present results do not exclude the possibility of more complex interactions between the receptors, or receptors and ligands; identification of their effects requires larger and more homogeneous cohorts.

## Materials and methods

### Study cohorts

The HSCT study population consisted of 1,491 HSCT donors from allogeneic HSCTs conducted in four countries: 765 from Finland (Helsinki and Turku University Hospitals), 352 from the UK (The Freeman Hospital, Newcastle Hospitals NHS Foundation Trust), 272 from Spain (IDIBGI Biobank), and 102 from Poland (Hematological departments of the Medical University of Gdansk, Maria Skłodowska-Curie Memorial Cancer Centre, and Institute of Oncology in Gliwice). The population demographics are described in Table 1.

The HSCT study population was subdivided into a discovery cohort of 1,045 HSCT donors and a replication cohort of 446 HSCT donors, and the discovery cohort included the earliest 2/3 of HSCTs from each population (the year of HSCT was among the covariates in statistical analyses). The study participants gave an informed consent, or when no longer possible, the permit was granted by VALVIRA, the National Supervisory Authority for Welfare and Health in Finland. All protocols, including the collection of samples and the use of these data, were approved by the Ethical Review Boards of each collaborating hospital. The permit numbers are V/74,832/2017 (Finland, VALVIRA), HUS/2152/2020 (Finland, Helsinki University Hospital), ETMK 78/2012 (Finland, Turku University Hospital), Biobank IDIBGI B.0000872 (Spain), 14/NE/1136 (the UK), KB-561/2019 (Poland). All protocols have been carried out according to relevant guidelines and regulation, such as the Declaration of Helsinki. In genome and statistical analyses, the SOPs of the R&D Department of the Finnish Red Cross Blood Service were followed. For example, only the pseudonyms for patients and donors were available to the study group and sensitive data was handled only in environments fulfilling the local regulations. No prisoners were recruited as donors.

In vitro NK cell cytotoxicity was determined from 14 blood donors who gave an informed consent to donate buffy coat from the standard blood donation bag for the present study. All 14 had genotypes available in the Blood Service Biobank. The buffy coat samples were collected and handled according to the ethical permit HUS/1854/2019 (Helsinki University Ethical Review Board, Finland) and the biobank project permit 003-2019 (Blood Service Biobank, Finland). The biobank collects samples only from the blood donors who have given a broad biobank consent according to the Finnish Biobank Act. The Biobank is supervised by the Finnish Medicinal Authority FIMEA.

### Genotyping, lift-over, and genotype imputation

The HSCT donors were genotyped at the genome-wide level using the following arrays: Illumina Immunochip v1, Immunoarray v2, Illumina Global Screening Array v2 or v3, at the Finnish Institute of Molecular Medicine, Helsinki, Finland, or using an exome sequencing pipeline^31^ at the McGill Genome Centre, Montreal, Canada. The genotyping data underwent a lift-over^32^ to the human reference genome build GRCh38/hg38, followed by genome-wide SNP imputation. In the lift-over and imputation^33^, the THL Biobank’s SISu v3 reference panel was used for the Finnish samples, and for the rest, a reference panel of the European samples of the 1000Genomes project, provided with the protocols, was used. The blood donors were originally genotyped by the FinnGen research project^34^.

### Association analysis

In total 1,638 genetic polymorphisms, located flanking or in 21 NK cell receptor genes were studied for their associations with HSCT outcome. The genes KLRD1 (CD94), ILT2 (LILRB1), ILT4 (LILRB2), NCR1 (NKp46), NCR2 (NKp44), NCR3 (NKp30), KLRC1 (NKG2A), KLRC2 (NKG2C), KLRK1 (NKG2D), ADGRG1 (GPR56), CD2, CD226 (DNAM-1), CD244, KLRG1, PD1, LAG3, Tim-3, CD160, PVRIG, CD96, and FCGR3A were selected from public databases based on their relevance in NK cell function, AML, hematological malignancies, or immunological diseases. A complete list of the polymorphisms and genes is provided in the Supplementary Material (Supplementary Table S1 online). The polymorphisms were filtered with pairwise linkage disequilibria (LD) using Plink 2.0^35^ with the parameters window size 500 kb and r^2 threshold 0.2. After imputation, filtering for common polymorphisms between the imputation reference panels, and LD-filtering, 1,638 polymorphisms remained. A workflow detailing all the analysis steps is depicted in Fig. 1.

Association analysis was conducted on the 1,638 NK cell receptor gene polymorphisms in the HSCT donors using acute GVHD (grade I-IV vs. grade 0, grade III-IV vs. grade 0), chronic GVHD (limited, extensive, grade unknown vs. grade 0; extensive vs. grade 0; extensive, grade unknown vs. grade 0), and relapse (yes vs. no) as endpoints. The HSCTs of nonmalignant disease were excluded from the analysis of relapse. The covariates included in the analyses were recipient and donor age, donor type (sibling, register, haploidentical), graft type, recipient–donor sex matching, conditioning regimen, disease (AML/myelodysplastic syndrome [MDS] or other), information on the occurrence of acute GVHD for the chronic GVHD endpoints, transplantation year, the country of origin, recipient–donor CMV combination, the presence of HLA-C1 and HLA-C2 alleles, recipient–donor HLA match score, and GvHD prophylaxis for the HSCTs. Missing donor and recipient ages were imputed with missForest the R package^36^ using the covariates recipient and donor age, donor type (sibling, register, haploidentical), graft type, recipient–donor sex matching, conditioning regimen, disease (AML/myelodysplastic syndrome [MDS] or other), information on the occurrence of acute GVHD for the chronic GVHD endpoints, transplantation year, and the country of origin.

The genotype probability of the imputed NK cell receptor gene polymorphisms was used as the dosage information in the association analysis. The analysis was performed with Plink 2.0^35^ with an additive model containing the parameters “covar-variance-standardize” to standardize quantitative covariates, and “ci 0.95” and “adjust” to compute the 95% confidence intervals and multiple testing-corrected p-values. First, all subpopulations were combined for the analysis, followed by investigating the effect sizes and their directions in the subpopulations separately. This was done both in the discovery and replication cohorts. The criteria for accepting an association for further analysis was a non-multiple testing-corrected p-value < 0.005 in the population combination in the discovery cohort.

### Survival analysis

We performed survival analyses on relapse-free survival for the polymorphisms associated with relapse in the discovery cohort. Kaplan-Meier analysis and log-rank tests were used *(ggsurvfit* the R package version 0.3.1), and further, we performed Cox regression adjusting the analysis with covariates (survival the R package, version 3.5-7). The covariates were recipient and donor age, donor type (sibling, register, haploidentical), graft type, recipient–donor sex matching, conditioning regimen, disease (AML/myelodysplastic syndrome [MDS] or other), information on the occurrence of acute GVHD for the chronic GVHD endpoints, transplantation year, the country of origin, recipient–donor CMV combination, the presence of HLA-C1 and HLA-C2 alleles, recipient–donor HLA match score, and GvHD prophylaxis.

### NK cell receptor gene polymorphism selection with lasso

In addition to selecting NK cell receptor gene polymorphisms for the cytotoxicity analysis based on the results of the genetic association analysis, we used lasso regression analysis with glmnet the R package^37^ for obtaining additional support for the selection. The same covariates and dosage information as in the association analysis were included in constructing the lasso models, and they were created separately for the six endpoints (acute GVHD (grade I-IV vs. grade 0, grade III-IV vs. grade 0), chronic GVHD (limited, extensive, grade unknown vs. grade 0; extensive vs. grade 0; extensive, grade unknown vs. grade 0), relapse (yes vs. no)) in the discovery and replication cohorts after which the variables included in the models were compared. The tuning parameter lambda was chosen with a ten-fold cross-validation for each model.

The best candidate NK cell receptor gene polymorphisms to assess for in the in vitro data were selected based on the HSCT association results and lasso models in the discovery cohorts. For the polymorphisms selected from the association analysis, we examined if lasso had selected them into its models for the respective endpoints.

### NK cell cytotoxic activity in vitro assays

Human peripheral blood mononuclear cells were obtained from blood donors using Ficoll-Paque™ Plus (GE Healthcare Life Sciences) density gradient centrifugation, following the manufacturer’s guidelines. To isolate NK cells, the samples were processed using the MicroBead technology (NK cell isolation kit, Miltenyi Biotec) and then cryopreserved for future analyses.

For cytotoxic analyses, NK cells were thawed and allowed to rest overnight in NK MACS Basal medium supplemented with NK MACS Medium Supplement (Miltenyi Biotec), 5% human AB serum (Seralab), and interleukins (IL-2, 500 IU/ml; IL-15 20 ng/ml, Research grade, Miltenyi Biotec). Luciferase-transduced K562-luc2 (ATCC^®^ CCL¬243¬LUC2™) cells were used as the target cells. To assess the cytotoxic efficacy, NK cells and K562-luc2 + target cells were co-cultured at various effector: target (E: T) ratios (0.25:1; 0.5:1; 1:1; 2:1; 4:1; 8:1) for 16–18 h. Luciferin reagent (ONE-Glo luciferase reagent, Promega, Madison, USA) was added to the co-cultures and the living target cells were quantified using the Victor Nivo^®^ multimode plate reader (Perkin Elmer).

The NK cell in vitro cytotoxicity was analyzed using a linear mixed-effect model with a random intercept using the lme4 R package^38^. The models included the effector: target ratios (0.25:1; 0.5:1; 1:1; 2:1; 4:1; 8:1), HLA-C genotype, KIR B content group, cytomegalovirus status (pos/neg), and the genotypes for the NK cell receptor gene polymorphisms selected with the genetic association analysis and lasso regression on HSCT donors, as well as a group effect term to connect technical replicates and samples for the random intercept. The test was run for each genotype separately and statistical significance was set at 0.05.

## Electronic supplementary material

Below is the link to the electronic supplementary material.

Supplementary Material 1

## Data Availability

The code for association testing, lasso regression, and analyzing the in vitro data are publicly available in GitHub (https://github.com/FRCBS/NK_receptors/). Individual-level genotype, laboratory, or clinical data of HSCT donors or blood donors are not publicly available due to restrictions set by the ethical permits but can be asked for relevant studies from the corresponding author: jukka.partanen@veripalvelu.fi. The blood donor data available can be inquired from the Blood Service Biobank at https://www.veripalvelu.fi/en/biobank/for-researchers/.
